# Basic Principles and Role of Endoscopic Ultrasound in Diagnosis and Differentiation of Pancreatic Cancer from Other Pancreatic Lesions: A Comprehensive Review of Endoscopic Ultrasound for Pancreatic Cancer

**DOI:** 10.3390/jcm13092599

**Published:** 2024-04-28

**Authors:** Dushyant Singh Dahiya, Yash R. Shah, Hassam Ali, Saurabh Chandan, Manesh Kumar Gangwani, Andrew Canakis, Daryl Ramai, Umar Hayat, Bhanu Siva Mohan Pinnam, Amna Iqbal, Sheza Malik, Sahib Singh, Fouad Jaber, Saqr Alsakarneh, Islam Mohamed, Meer Akbar Ali, Mohammad Al-Haddad, Sumant Inamdar

**Affiliations:** 1 Division of Gastroenterology, Hepatology and Motility, The University of Kansas School of Medicine, Kansas City, KS 66160, USA; 2 Department of Internal Medicine, Trinity Health Oakland/Wayne State University, Pontiac, MI 48341, USA; 3 Division of Gastroenterology, Hepatology & Nutrition, East Carolina University/Brody School of Medicine, Greenville, NC 27858, USA; 4 Division of Gastroenterology and Hepatology, Creighton University School of Medicine, Omaha, NE 68178, USA; 5 Department of Gastroenterology and Hepatology, University of Arkansas for Medical Sciences, Little Rock, AR 72205, USA; 6 Division of Gastroenterology and Hepatology, University of Maryland School of Medicine, Baltimore, MD 21201, USA; 7 Division of Gastroenterology and Hepatology, The University of Utah School of Medicine, Salt Lake City, UT 84132, USA; 8 Department of Internal Medicine, Geisinger Wyoming Valley Medical Center, Wilkes Barre, PA 18711, USA; 9 Department of Internal Medicine, John H. Stroger Hospital of Cook County, Chicago, IL 60612, USA; 10 Department of Internal Medicine, University of Toledo Medical Center, Toledo, OH 43614, USA; 11 Department of Internal Medicine, Rochester General Hospital, Rochester, NY 14621, USA; 12 Department of Internal Medicine, Sinai Hospital, Baltimore, MD 21215, USA; 13 Section of Gastroenterology and Hepatology, Baylor College of Medicine, Houston, TX 77030, USA; 14 Department of Internal Medicine, University of Missouri-Kansas City, Kansas City, MO 64110, USA; 15 Division of Hepatology, University of Missouri School of Medicine, Columbia, MO 64108, USA; 16 Division of Gastroenterology and Hepatology, University of Jordan, 11942 Amman, Jordan; 17 Division of Gastroenterology and Hepatology, Indiana University School of Medicine, Indianapolis, IN 46202, USA

**Keywords:** pancreatic cystic lesions, endoscopic ultrasound, endoscopic interventions, advantages, disadvantages, artificial intelligence

## Abstract

Pancreatic cancer is one of the leading causes of cancer-related deaths worldwide. Pancreatic lesions consist of both neoplastic and non-neoplastic lesions and often pose a diagnostic and therapeutic challenge due to similar clinical and radiological features. In recent years, pancreatic lesions have been discovered more frequently as incidental findings due to the increased utilization and widespread availability of abdominal cross-sectional imaging. Therefore, it becomes imperative to establish an early and appropriate diagnosis with meticulous differentiation in an attempt to balance unnecessary treatment of benign pancreatic lesions and missing the opportunity for early intervention in malignant lesions. Endoscopic ultrasound (EUS) has become an important diagnostic modality for the identification and risk stratification of pancreatic lesions due to its ability to provide detailed imaging and acquisition of tissue samples for analysis with the help of fine-needle aspiration/biopsy. The recent development of EUS-based technology, including contrast-enhanced endoscopic ultrasound, real-time elastography–endoscopic ultrasound, miniature probe ultrasound, confocal laser endomicroscopy, and the application of artificial intelligence has significantly augmented the diagnostic accuracy of EUS as it enables better evaluation of the number, location, dimension, wall thickness, and contents of these lesions. This article provides a comprehensive overview of the role of the different types of EUS available for the diagnosis and differentiation of pancreatic cancer from other pancreatic lesions while discussing their key strengths and important limitations.

## 1. Introduction

Pancreatic cancer is the fourth leading cause of cancer-related deaths in the United States (US), with an estimated 50,500 deaths in 2023 [1,2]. It is imperative to detect pancreatic cancer early due to its highly aggressive nature and propensity for early metastasis. By the time a patient develops clinical signs and symptoms, in approximately 80–90% of cases, it is usually unresectable [3,4].

In current clinical practice, pancreatic lesions are being increasingly diagnosed due to the widespread availability and utilization of high-resolution imaging such as computed tomography (CT), magnetic resonance imaging (MRI), and cross-sectional abdominal ultrasound (US) [5]. The incidental detection of pancreatic cystic lesions (PCLs) alone is estimated to range from 0.5 to 45% [6]. However, pancreatic lesions encompass a wide spectrum of lesions, including not only benign cysts but also neuroendocrine tumors and pancreatic adenocarcinoma [7]. It is vital to distinguish neoplastic from non-neoplastic lesions, as well as non-mucinous cysts from mucinous cysts, due to an increased risk of malignant conversion of the latter [7,8,9,10,11]. Early accurate diagnosis and intervention of neoplastic lesions may significantly impact clinical outcomes. The classifications of pancreatic lesions that are important to differentiate from pancreatic cancer are outlined in Table 1 [7,8,9,10,11].

Endoscopic ultrasound (EUS) has emerged as a pivotal tool in the diagnostic and therapeutic landscape of pancreatic diseases [11]. It has evolved from a mainly diagnostic modality to one that can facilitate tissue diagnosis with the help of fine-needle aspiration (FNA) and fine-needle biopsy (FNB), allowing better histological characterization of the pancreatic lesions [12]. Its minimally invasive nature and the ability to obtain higher-resolution images have made EUS indispensable, particularly in detecting smaller pancreatic lesions [13]. Furthermore, compared to traditional cross-sectional imaging, it can better assess the size, shape, number of cysts, presence or absence of septations, solid and cystic components, and pancreatic ductal diameter and provide a detailed evaluation of nearby lymph nodes [13]. Additionally, the continuous evolution of EUS over the years, including the development of real-time elastography (RTE-EUS), contrast-enhanced EUS (CE-EUS), EUS-guided fine-needle aspiration (EUS-FNA), EUS-guided fine-needle biopsy (FNB), and the EUS-guided rendezvous technique (EUS-RV) has expanded its therapeutic potential, enhanced application, and enables therapeutic endoscopists to establish a highly accurate diagnosis, thereby revolutionizing patient care [14,15,16,17].

In this comprehensive review, we discuss the pivotal multidimensional role of EUS in the management of pancreatic lesions and the differentiation of pancreatic cancer from other lesions. Furthermore, we also examine and compare the diagnostic accuracy of various EUS techniques for pancreatic lesions—where traditional imaging techniques fall short. This article further highlights the invaluable contribution of EUS and outlines the areas of future development of EUS for the evaluation of pancreatic lesions.

## 2. Endoscopic Ultrasound: Basic Principles

EUS was developed in the early 1980s for better visualization of the pancreaticobiliary system as conventional ultrasound imaging of these deeper structures was limited due to overlying bowel [18]. EUS combines two basic modalities—endoscopy, which aids in luminal visualization, and high-frequency acoustic waves, which are utilized to image parts of the gastrointestinal tract, internal organs, blood vessels, and lymph nodes in its proximity [19,20].

The distal tip of the EUS endoscope consists of transducers and receivers that produce ultrasonic waves and receive waves reflected off the tissue, respectively, and generate 2D images by processing electrical signals [20]. The image quality depends on the transmission power, which is defined as the energy per unit of time acting on the insonated tissue [21]. Axial resolution is determined by the ultrasound pulse length, which is directly proportional to the frequency; however, tissue penetration decreases with increased frequency, thereby limiting the depth of tissue penetration [20]. The lateral resolution is a function of the width of each ultrasound wave (or beam), and its resolution is the best at the narrowest portion, called the focal zone [16]. Transducer size and frequency ultimately determine the shape and dimension of the beam [20].

From a procedural standpoint, EUS is performed in a similar fashion as standard endoscopy and can be performed on an outpatient basis under intravenous sedation [22]. However, the procedure is operator-dependent, and the experience of the therapeutic endoscopist performing the procedure is directly proportional to the quality of the examination. The duration of the procedure depends on the complexity of the area being imaged, the indication of the study, the operator’s experience, and the need to obtain tissue samples via FNA/FNB [22].

Conventionally, there are two types of EUS endoscopes, namely, the radial and the linear/convex endoscope. Both these endoscopes provide views in a plane parallel to the scope shaft [23]. During the procedure, the EUS endoscope is passed through the mouth until the tip reaches the potential area of interest. Extraluminal lesions are assessed using specific anatomical stations [22,23]. The three main stations for imaging the pancreaticobiliary tract are the stomach, duodenal bulb, and second portion of the duodenum [23]. The pancreatic body and tail, spleen, lymph nodes, left adrenal gland, and left lobe of the liver can be visualized through the gastric wall [22]. The aorta and the celiac artery, along with the superior mesenteric artery, can be visualized when the scope is rotated clockwise in this location [23]. The pancreatic body and tail can be visualized by further clockwise rotation of the scope [23]. The portal vein and the pancreatic head can be visualized through the duodenal bulb station, and the bile duct can be seen running parallel to the portal vein [23]. As the endoscope passes into the descending part of the duodenum, the pancreatic head, ampulla of Vater, and the uncinate can be clearly visualized [22,23].

## 3. Conventional (Radial and Linear) Endoscopic Ultrasound

Radial EUS was the first to be developed and available commercially. It consists of a rotating ultrasound transducer situated distal to the oblique-viewing lens at the tip of the endoscope with a range of frequencies between 5 and 20 MHz, offering a 360-degree view [19,22]. The water-filled balloon at the end of the endoscope enables acoustic coupling, and the images obtained with the radial EUS are cross-sectional and perpendicular to the endoscope shaft (similar to the images obtained via a CT scan) [22]. Radial EUS is only useful for staging since it does not have a working channel. This is because the needle, through the working channel, would only appear as a dot in the radial echoendoscope as the ultrasound beam passes through the needle and at right angles [22,24]. On the other hand, the plane of the linear EUS ranges from 120 to 180 degrees, as the scanning plane is on the same axis as the scope shaft and the accessory channel [22]. In the linear EUS endoscope, the needle can be passed through the accessory channel and is visible in its entirety as it passes along the same axis as the ultrasound beam, enabling real-time guidance for needle-based interventions [22,24].

**Figure 1:**
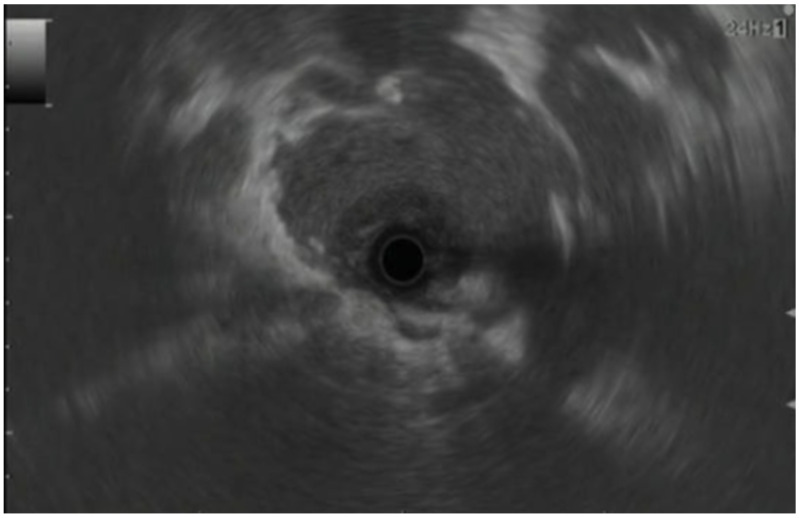
EUS showing pancreatic cancer of head of pancreas [25].

In EUS, pancreatic adenocarcinoma appears as an irregular hypoechoic mass in the pancreatic parenchyma with poorly defined margins, pancreatic duct dilation, parenchymal atrophy (in advanced cases), and an absence of cysts within the mass (Figure 1) [25]. Lymphadenopathy and vascular invasion may also be noted [26,27]. Prior published literature has reported a higher degree of accuracy of conventional EUS techniques for diagnosing pancreatic adenocarcinoma compared to traditional cross-sectional imaging. A study by Rivadeneira et al. that compared linear EUS with CT scanning for staging of periampullary tumors demonstrated the sensitivity, specificity, and accuracy of EUS to be 100%, 75%, and 89%, compared to 68%, 50%, and 67%, respectively, for CT scanning [28]. A recent meta-analysis by Kitano et al., which included 22 studies with 1170 patients, noted that the median sensitivity of EUS for the detection of pancreatic tumors was 94% [29]. Furthermore, upon including 19 studies with a direct comparison of EUS and CT imaging, the authors noted that the sensitivity of EUS (98%) was far superior to that of CT scanning (74%) [29]. For the detection of smaller pancreatic tumors, especially those <30 mm in diameter, Muller et al. noted that EUS was far more sensitive (93%) compared to CT scanning (53%) and MRI (67%) [30]. Additionally, the specificity and accuracy for EUS were also higher at 100% and 96% compared to 64% and 67% for CT scanning and 100% and 84% for MRI, respectively [30].

EUS has also found great success in the diagnosis of pancreatic neuroendocrine tumors. A study by Deguelte et al. showed that EUS is the most sensitive test, with a detection rate of 86% for neuroendocrine tumors [31]. Therefore, it has been recommended for surveillance in patients with multiple endocrine neoplasia type 1 [31]. Neuroendocrine tumors will typically enhance for all imaging modalities due to their rich vascularization and appear similar to arterial enhancement during CE-EUS [31].

Conventional EUS has also found application in differentiating pancreatic cancer from other non-malignant causes that share similar radiological features of cross-sectional imaging. Not only does EUS help identify pancreatic cysts >30 mm, but it can also detect the presence of masses within surrounding tissue and dilatation of the Wirsung duct, both of which are features of an underlying malignancy [32]. Furthermore, EUS can also help differentiate autoimmune and chronic pancreatitis from pancreatic cancer, thereby limiting unnecessary intervention. In EUS imaging, autoimmune pancreatitis appears as a hypoechoic area with diffuse enlargement, bile duct wall thickening, and hypoechoic peripancreatic margins, which are in stark contrast to pancreatic cancer [33]. The Rosemont Criteria can help differentiate chronic pancreatitis from pancreatic cancer [34]. The major criteria include the presence of hyperechoic areas with shadowing, lobularity with honeycombing, and the presence of main pancreatic duct calculi [34]. Meanwhile, the minor criteria include cysts, dilated ducts >3.5 mm, hyperechoic duct walls, non-shadowing hyperechoic foci, and lobularity with non-contiguous lobules [34].

**Figure 2:**
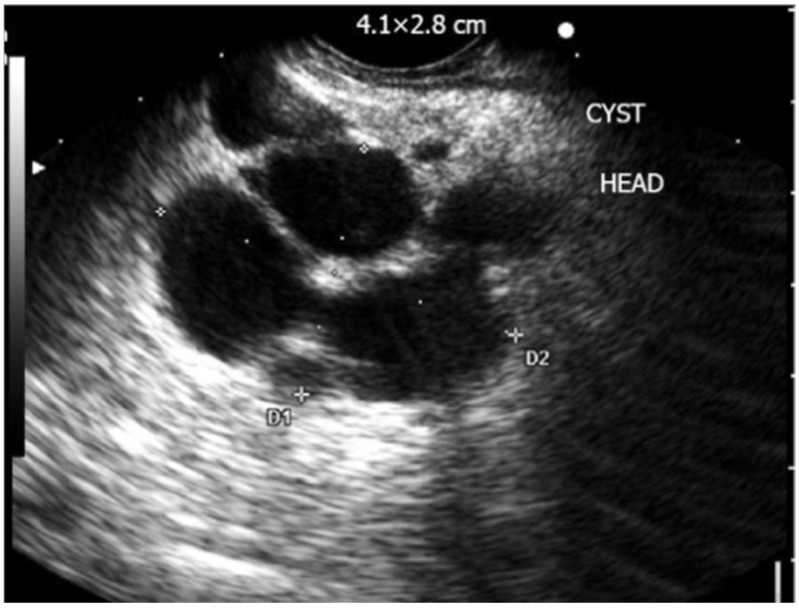
EUS showing complex multilocular cyst consistent with intraductal papillary mucinous neoplasm (D1 and D2: Dimensions of lesion) [35].

**Figure 3:**
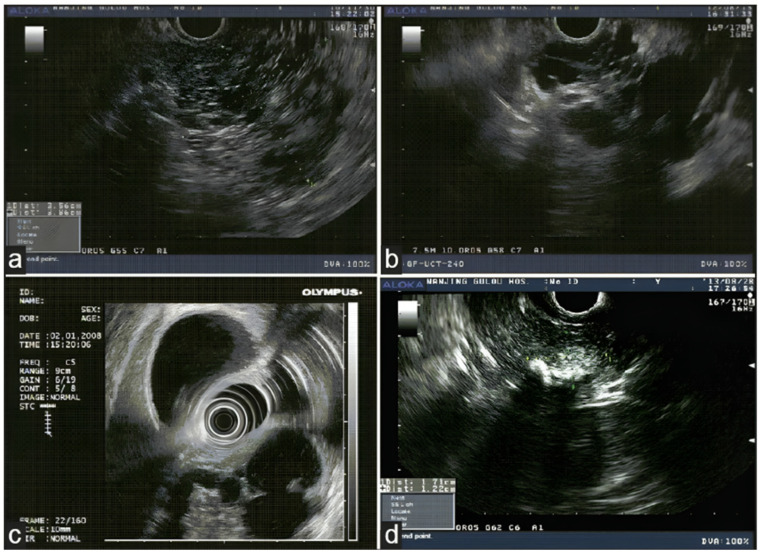
Morphologic features of various PCLs in EUS: (**a**) microcystic serous adenoma with honeycombing appearance in the pancreas, (**b**) mucinous cystic neoplasm with septations in the head of the pancreas, (**c**) intraductal papillary mucinous neoplasm in the head of the pancreas, and (**d**) solid pseudopapillary neoplasm as mixture echo mass with calcifications in the head of the pancreas [36].

Figure 2 shows the features of intraductal papillary mucinous neoplasm, and Figure 3 shows the morphological features of some of the pancreatic lesions in EUS [35,36]. The characteristic features of various commonly encountered pathologies in EUS are discussed in Table 2 [37,38,39,40,41,42,43].

## 4. Miniature Probe Endoscopic Ultrasound

Conventional EUS using dedicated endoscopes with a frequency from 7.7 to 12 MHz has several drawbacks. One key limitation is difficulty in passing the endoscope through severe strictures (larger diameter of the endoscope measuring 12–13 mm), which makes imaging of certain areas of the pancreaticobiliary tract difficult, and the image resolution for small lesions in these areas may also be unsatisfactory [44]. Miniprobe EUS, which was about 2 mm in diameter with a frequency ranging from 12 to 20 MHz, was developed specifically to overcome this limitation. The miniprobe EUS can be passed through the working channels of the standard endoscope and provides high-resolution images of the area of interest [44].

Along with maneuverability, the diagnostic accuracy of miniprobe EUS has also been proven to be superior to conventional EUS for specific parts of the gastrointestinal tract and the pancreaticobiliary system [44]. The literature has proven that the evaluation of a few gastrointestinal pathologies with miniprobe EUS has provided additional key information, which eventually led to changes in treatment plans [45,46,47,48]. A study by Kanemaki et al. on 26 patients who underwent 3D intraductal ultrasound using miniprobe EUS demonstrated that it was highly effective for accurate assessment of tumor extension, staging, and the relationship with surrounding structures [49].

A major drawback of the miniprobe EUS is its lifespan, which is about 50–100 procedures. Although it is preferred for quick and easy imaging of areas that are hard to visualize on conventional EUS, in the era of ‘green endoscopy’, it may not be the most viable option [50]. Furthermore, its fragility and high cost also limit widespread application [50].

## 5. Real-Time Elastography–Endoscopic Ultrasound

Elastography, a non-invasive tool that can measure tissue stiffness, has been used with EUS as an integrated software [51,52]. The use of EUS elastography for the evaluation of the pancreas was first reported in 2006 [53]. EUS elastography comprises the following two types:

1. Strain Elastography (Qualitative Elastography): It estimates the stiffness of the tissue by measuring the degree of strain [29,52]. The results are interpreted with the help of a colored scale, wherein red-green areas indicate softer tissues and blue areas indicate stiffer tissues [29,52]. The main limitations of qualitative elastography are that it lacks reproducibility as the interpretation of the colors is highly operator-dependent, and it provides limited information when comparing results for different patients and lesions [54,55].
2. Shear-Wave Elastography (Quantitative Elastography): It measures tissue stiffness by measuring the propagation of the shear waves, which is the emission of focused waves from the probe to the target lesion, also known as acoustic radiation force impulse [29]. Another method of shear-wave elastography is the semi-quantitative analysis, which allows for the measurement of tissue stiffness by calculating the strain ratio (ratio of stiffness of area of interest on the target tissue and smaller region of interest of a reference tissue) and strain histogram technique where an average hue histogram represents the colors and thereby the stiffness of the tissue [56]. It is important to note that only strain elastography is available with EUS for the evaluation and characterization of pancreatic lesions [29].

**Figure 4:**
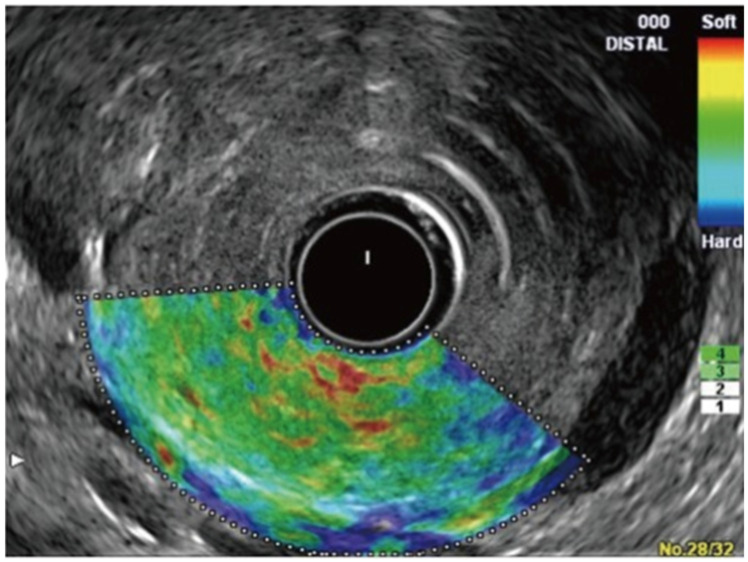
EUS elastography of normal pancreas showing uniform, homogenous, green color distribution (intermediate stiffness) [57].

**Figure 5:**
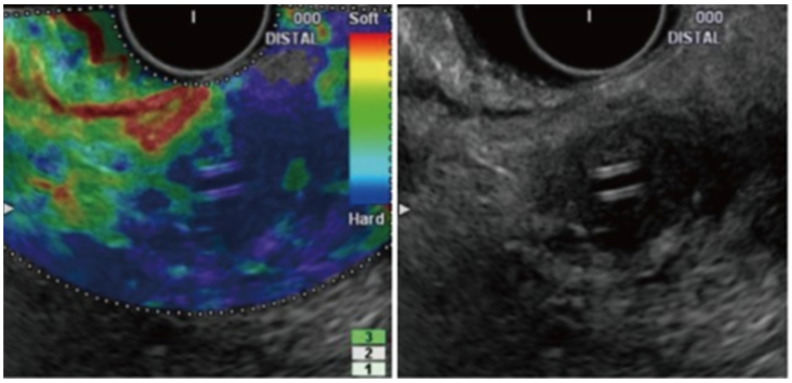
EUS elastography of pancreatic cancer showing heterogeneous blue color distribution representing hard stiffness [57].

The difference between normal pancreatic tissue and pancreatic cancer in EUS elastography is shown in Figure 4 and Figure 5, respectively [57].

A prospective study on 78 patients by Iglesias-Garcia et al., which utilized EUS elastography, demonstrated that the green predominant pattern in either homogenous or heterogenous lesions was highly accurate in excluding malignancy, and the homogeneous or heterogeneous blue predominant pattern was suggestive of a malignant tumor with an overall sensitivity, specificity, positive predictive value, negative predictive value, and accuracy of 100%, 85.5%, 90.7%, 100%, and 94%, respectively [58]. The authors noted that the mean strain was 1.68 (95% CI 1.59–1.78) for normal tissue, 3.28 (95% CI 2.61–3.96) for inflammatory masses, and 18.12 (95% CI 16.03–20.21) for pancreatic adenocarcinoma [58]. A meta-analysis by Kitnao et al. that included 1568 patients across 15 studies observed an overall sensitivity and specificity for EUS elastography of 93% and 63%, respectively, for solid pancreatic masses [29]. Another meta-analysis by Pei et al. that included 1042 patients across 13 studies showed that the pooled sensitivity and specificity for EUS elastography in differentiating benign and malignant solid pancreatic masses are 95% and 69%, respectively, with color pattern and blinding associated with heterogeneity [59]. Furthermore, a study by Kim et al. on the optimal cut-off value of the strain ratio in different pancreatic etiologies like normal pancreas, chronic pancreatitis, and pancreatic cancer showed that the mean strain ratio was 3.78 ± 1.35 for normal pancreas, 8.21 ± 5.16 for chronic pancreatitis, and 21.80 ± 12.23 for pancreatic cancer with a sensitivity, specificity, and accuracy of 71.6%, 75.2%, and 74.8%, respectively, for detecting chronic pancreatitis and 95.6%, 96.3%, and 96.2%, respectively, for detecting pancreatic cancer [60]. Hence, EUS elastography is an important tool in the arsenal of therapeutic endoscopists.

## 6. Contrast-Enhanced Endoscopic Ultrasound

CE-EUS was first described by Kato et al., who was the first to perform extracorporeal ultrasonographic angiography and EUS angiography for pancreatic lesions [61]. Since then, there has been significant development in CE-EUS with the use of innovative contrast agents, primarily composed of hexafluoride microbubbles, leading to a cost-effective, fast, and simple examination [61,62]. Other contrast agents used in CE-EUS include octafluoropropane or perfluorobutane (available only in Europe, Norway, and Denmark at present) [63,64].

**Figure 6:**
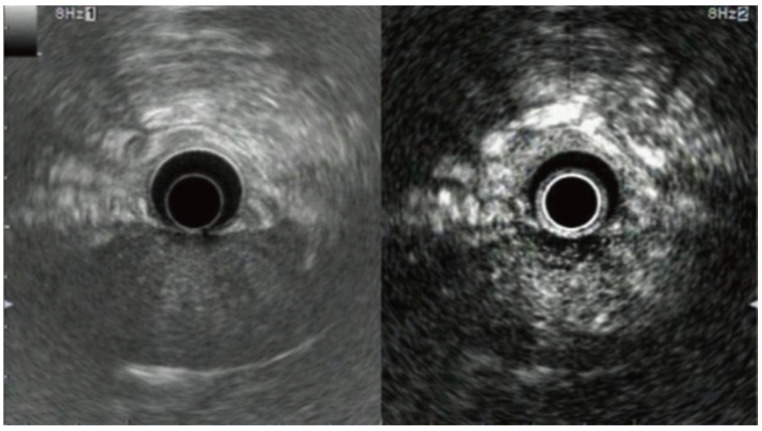
Difference between EUS and CE-EUS in pancreatitis. Left: conventional EUS; slightly hypoechoic area without a clear margin at pancreas head. Right: CE-EUS showing enhancement in the area with pancreatitis as compared to normal tissue with a clear margin [68].

The linear-array EUS endoscope with a frequency of 7.5–10 MHz is most frequently used during CE-EUS as it can also be used for FNA to obtain tissue samples [65,66]. There are two main techniques of CE-EUS, which include contrast-enhanced endoscopic Doppler ultrasound with a high mechanical index (CHEMI-EUS) and contrast-enhanced low mechanical index EUS (CLEMI-EUS) [14]. During the procedure, when the EUS probe is near the area of interest, a bolus of microbubble followed by normal saline flush is injected into the patient [67]. The ultrasound processor uses a specific software that filters background tissue signals and produces only contrast-enhanced images [67]. Furthermore, as the contrast microbubbles have a small diameter (2.5 μm), their distribution is purely intravascular, enabling clear visualization of small blood vessels and high-resolution visualization of pancreatic parenchyma [67]. The difference between conventional EUS and CE-EUS is shown in Figure 6 [68].

**Figure 7:**
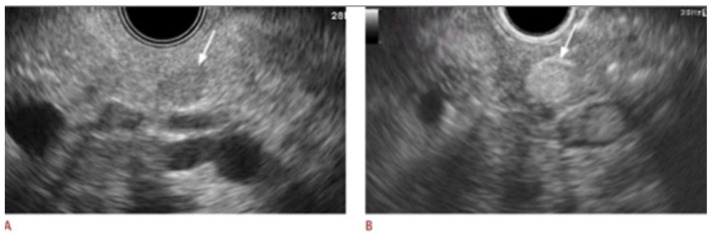
Neuroendocrine tumor in EUS and CE-EUS: (**A**) Conventional EUS showing hypoechoic tumor in the body of pancreas (white arrow). (**B**) CE-EUS showing hyperenhancement of the lesion (white arrow) [70].

Numerous cohort studies have assessed the utilization of CE-EUS for various pancreatic lesions. A study by Kitano et al. tested the diagnostic accuracy of CE-EUS in 277 patients and noted that the sensitivity and specificity of CE-EUS in diagnosing ductal carcinoma were 95.1% and 89%, respectively, and for small carcinoma, they were 91.2% and 94.4%, respectively [69]. Additionally, CE-EUS was also noted to have a sensitivity and specificity of 78.9% and 98.7%, respectively, in detecting neuroendocrine tumors [69]. Figure 7 highlights the difference between conventional EUS and CE-EUS for neuroendocrine tumors [70]. Another study by Fusaroli et al. demonstrated that the sensitivity, specificity, and accuracy of CE-EUS for hypoenhancing lesions, the majority of which were pancreatic adenocarcinomas, were 96%, 64%, and 82%, respectively, compared to 86%, 18%, and 57%, respectively, in conventional EUS [71]. Moreover, for hyperenhancing lesions, the sensitivity, specificity, and accuracy of CE-EUS in excluding adenocarcinoma were 39%, 98%, and 72%, respectively, and for predicting these lesions as neuroendocrine tumors, they were 69%, 90%, and 88%, respectively [71].

Multiple pooled analyses investigating the usefulness of CE-EUS for pancreatic lesions have also yielded favorable results. A meta-analysis by Yamashita et al. that assessed the usefulness of CE-EUS with enhancement patterns noted that the pooled sensitivity and specificity of CE-EUS were 93% and 80%, respectively, whereas the meta-analysis by Brand et al. observed that the sensitivity and specificity of EUS alone were 93% and 55%, respectively, for establishing a diagnosis of pancreatic cancer [72,73]. CE-EUS was also found to be helpful in differentiating malignant pancreatic cystic lesions with a sensitivity of 100% and specificity of 80–89%, as it distinguishes mural nodules in the intrapapillary mucinous neoplasm [74]. The meta-analysis by Lisotti et al. (10 studies with 532 patients) which evaluated the pooled diagnostic performance of CE-EUS in characterization of mural nodules within PCL demonstrated a pooled sensitivity of 88.2% (95% CI: 82.7–92.5%), a specificity of 79.1% (95% CI: 74.5–83.3%), and a diagnostic accuracy of 89.6% (95% CI: 83.4–95.8%) [75].

Obtaining tissue samples via CE-EUS-FNA has also been shown to be superior compared to conventional EUS-FNA. In a meta-analysis of six studies (701 patients), the authors observed that the CE-EUS-FNA group had a pooled diagnostic sensitivity of 84.6% (95% CI 80.7–88.6%), compared to 75.3% (95% CI 67–83.5%) in the EUS-FNA group (odds ratio 1.74, 95% CI 1.26–2.40; *p* < 0.001) [76]. Furthermore, the pooled sample accuracy (OR 1.52, 95% CI 1.01–2.31; *p* = 0.05) and pooled sample adequacy (OR 2.40, 95% CI 1.38–4.17; *p* = 0.02) of CE-EUS-FNA is also higher as compared to conventional EUS-FNA [76]. Hence, this makes CE-EUS-FNA more desirable for tissue acquisition compared to conventional EUS-FNA.

## 7. Endoscopic Ultrasound Fine-Needle Aspiration and Biopsy

EUS-FNA and EUS-FNB are both EUS-guided tissue acquisition techniques that are considered highly safe and are of excellent diagnostic value in the evaluation of pancreatic mass lesions and subepithelial lesions and for lymph node biopsy [15].

### 7.1. Endoscopic Ultrasound Fine-Needle Aspiration

EUS-FNA, first introduced in 1992, is currently the recommended standard of care by the American Society of Gastrointestinal Endoscopy (ASGE) and the European Society of Gastrointestinal Endoscopy (ESGE) for sampling pancreatic solid masses, subepithelial lesions, and lymph nodes [15,77,78]. The diagnostic ability of EUS-FNA is supplemented by rapid onsite evaluation of the acquired tissue sample, which further augments its diagnostic accuracy [79,80,81]. Depending on the size, location, and type of the lesion, various needle sizes are available for EUS-FNA [7,15]. For example, the 19G needle is useful for lesions located in the pancreatic tail or body, 19G flexible or 22G for lesions in the pancreatic head or uncinate process, and 25G only for clear solid lesions [7,82]. However, a meta-analysis of seven clinical trials (732 pancreatic lesions) by Facciorusso et al. showed non-superiority of 25G compared to 22G needle for tissue sampling of solid pancreatic masses (*p*-value = 0.13) [83]. Furthermore, the authors also did not find a difference in specificity between the two groups (*p*-value = 0.85) [83].

Over time, the landscape of diagnosing pancreatic lesions has evolved from morphological assessment to analysis of contents, i.e., the cystic fluid. This fluid acquired during EUS-FNA can be checked for amylase, carcinoembryogenic antigen (CEA), CA 19-9, glucose, and cellularity. Commonly used techniques for aspiration of cystic fluid include negative pressure suction or slow stylet pull [78,84,85]. In current literature, EUS-FNA reportedly has a diagnostic accuracy ranging from 77 to 95% for pancreatic masses [78,84,85]. A meta-analysis by Banafea et al. consisting of 22 studies assessing the overall performance of EUA-FNA in the diagnosis of solid pancreatic lesions noted that the pooled sensitivity and specificity of EUS-FNA were 90.8% (95% CI: 89.4–92.0%) and 96.5% (95% CI 94.8–97.7%), respectively, with an overall diagnostic accuracy of 91% [86]. Additionally, the positive and negative likelihood ratios were 14.80 (95% CI, 8.00–27.30) and 0.12 (95% CI, 0.09–0.16), respectively [86]. The low negative likelihood ratio potentially limits the use of EUS-FNA in pancreatic cancer detection as it may miss early resectable tumors [86]. Interestingly, the sensitivity and specificity of EUS-FNA for the detection of malignancy in patients with chronic pancreatitis (54% and 73.4%, respectively) were found to be lower as compared to that for normal pancreatic tissue (89.0% and 91.3%, respectively) [87]. Furthermore, as discussed earlier, the diagnostic yield increases by 10–30% with the concomitant use of rapid onsite evaluation with reported accuracy, sensitivity, and specificity ranging from 93.3 to 96.8%, 88.6 to 96.2%, and 99 to 100%, respectively [88,89,90].

The overall complication rate of EUS-FNA has been estimated to be approximately 2.5%, highlighting the excellent safety profile of the procedure [91]. Reported complications of the procedure include acute pancreatitis, infections, intestinal perforation, and malignant seeding while obtaining tissue samples [91]. A multicenter retrospective analysis consisting of 506 patients who underwent EUS-guided through the needle biopsy of PCLs showed that age (OR: 1.32 95% CI: 1.09–2.14; *p*-value = 0.05), number of TTNB passes (OR from 2.17, 1.32–4.34 to OR 3.16, 2.03–6.34 with the increase in the number of passes), complete aspiration of the cyst (OR 0.56, 0.31–0.95; *p*-value = 0.02), and diagnosis of intraductal papillary mucinous neoplasm (OR 4.16, 2.27–7.69; *p* < 0.001) were independent predictors of adverse events [92]. Furthermore, despite many advancements, the role of FNA is limited due to low tissue acquisition, an inability to obtain core tissue samples with preserved architecture, thereby making immunohistochemical staining and histologic diagnosis difficult, and a lack of widespread availability of therapeutic endoscopists skilled at performing the procedure [93,94].

### 7.2. Endoscopic Ultrasound Fine-Needle Biopsy

EUS–FNB was developed to obtain tissue samples that would enable pathologists to perform immunohistochemical staining, thereby overcoming a key limitation of EUS-FNA [15]. A 19G Trucut needle biopsy with a penetrating stylet was eventually developed, and initial results were promising compared to EUS-FNA [15,95]. However, its use was fairly limited due to mechanical failure in areas that require an angulated endoscope, such as the duodenum [15,95]. Later, a second-generation core biopsy needle like ProCore (Cook Endoscopy), which was equipped with a reverse bevel for tissue acquisition, and SharkCore (Medtronic Corp., Minneapolis, MN, USA), which was equipped with a fork tip, were developed for better tissue acquisition in an attempt to improve diagnostic yield [93]. The development of second-generation techniques has allowed immunohistochemistry, which is required for the diagnosis of etiologies such as autoimmune pancreatitis, lymphoma, and metastasis, and for molecular analysis of pancreatic malignancies [96,97].

EUS-FNB has a high diagnostic accuracy for pancreatic malignancy as it enables tissue acquisition for molecular profiling and histological analysis [1]. A multicenter randomized controlled trial by van Riet et al. comparing EUS-FNA and EUS-FNB demonstrated that EUS-FNB had a higher histologic yield (82% vs. 72%; *p* = 0.002), accuracy for diagnosing malignancy (87% vs. 78%, *p* = 0.002), and Bethesda classification (82% vs. 72%, *p* = 0.002) compared to EUS-FNA [98]. Additionally, the authors noted a higher odds ratio (3.53; 95% CI, 1.55–8.56; *p* = 0.004) when corrected for indication for the procedure, size of the lesion, total number of passes, and onsite pathologist [98].

The indications for EUS for PCL with high-risk features by various surveillance guidelines are listed in Table 3 [11,99,100,101,102].

## 8. Cost-Effectiveness of EUS in Evaluation of Pancreatic Lesions

A combination of different diagnostic modalities is often utilized for the evaluation of pancreatic lesions. It is vital to assess the cost-effectiveness of these EUS modalities. While there are similarities between surveillance guidelines by different international/national societies, each varies significantly in the frequency of imaging, type of imaging, and the threshold for EUS and surgery. The study by Faccioli et al. demonstrated that follow-up of PCL by CE-EUS is more cost-effective as compared to the Fukuoka Guidelines and the Italian Guidelines, with savings of EUR 832.27 (54.13%) and EUR 12.22 (14.87%), respectively, for evaluation of branch duct intrapapillary mucinous neoplasms (IPMNs) <1 cm, savings of EUR 276.32 (15.73%) and EUR 183.45 (11.02%), respectively, for branch duct intrapapillary mucinous neoplasms of 1–2 cm, and savings of EUR 5516 (66.71%) and EUR 1640.2 (37.34%), respectively, for branch duct intrapapillary mucinous neoplasms of 2–3 cm [103]. CE-EUS was more cost-effective by EUR 5162.39 (58.35%) in the follow-up of mucinous cystic neoplasm compared to the American College of Gastroenterology and European evidence-based guidelines [103]. The follow-up of serous cystic neoplasm <4 cm with CE-EUS was also proven to be more cost-effective by EUR 894.66 (40.73%) and EUR 321 (19.78%) as compared to European evidence-based guidelines and Italian guidelines follow-up [103]. The study by Lobo et al. compared the cost-effectiveness of the 2015 American Gastroenterological Association Guidelines with the 2017 International Consensus Guidelines for PCL [104]. The authors noted that more imaging studies (116,997 vs. 68,912) and more surgeries (711 vs. 163) led to higher total costs (USD 168.3 million vs. USD 89.4 million) with a similar number of deaths in the consensus compared to the American Gastroenterological Association Guidelines [100,102,104]. A study by Kumar et al. on the cost-effectiveness of EUS for pancreatic cancer screening in high-risk individuals showed that EUS is cost-effective in patients with a lifetime risk of pancreatic cancer greater than 108% or at lower probabilities if the life expectancy was at least 16 years after resection of the lesion with missed lesion rates of <5% on index EUS [105].

It is important to understand that the primary goal of surveillance of pancreatic lesions is to detect a potential preventable or curable malignancy while carefully assessing the risks, cost-effectiveness, and associated morbidity and mortality [38]. While a less intensive strategy may be followed for patients with less worrisome lesion features, more aggressive strategies should be implemented for patients with high-risk features like the presence of a mural nodule or solid component, dilatation of the pancreatic duct, pancreatic cystic lesion of size ≥3–4 cm, and positive cytology on PCL fluid aspiration [38].

## 9. Future Innovations in Endoscopic Ultrasound

Given current limitations, there are significant opportunities for technological advancements in EUS to improve diagnostic accuracy and aid in the management of pancreatic lesions. Confocal laser endomicroscopy and DNA analysis are the two emerging innovative techniques that will revolutionize traditional EUS.

EUS-guided confocal laser endomicroscopy (EUS-CLE) allows visualization of the epithelial lining inside the pancreatic cyst and the vascular structures supplying the cyst [106]. Numerous studies have been conducted successfully to assess the effectiveness of EUS-CLE, particularly for the differentiation of PCLs. The CONTACT-2 study in 2018 noted that the sensitivity, specificity, positive predictive value, and negative predictive value of EUS-CLE are 96%, 95%, 98% and 91%, respectively, for the assessment of various premalignant PCLs like mucinous cystic neoplasm, intrapapillary mucinous cyst, cystic neuroendocrine tumor, and cystic lymphomas [107]. A meta-analysis by Kovacevic et al. comparing EUS-guided biopsy and EUS-CLE showed similar technical success, diagnostic performance, and safety profile of the two procedures, but the diagnostic yield of EUS-CLE was significantly higher [108]. However, EUS-CLE has some key limitations. The price of a single EUS-CLE system is approximately USD 100,000, which is much higher than that of an EUS through-the-needle forceps biopsy, costing about USD 400 per examination [109]. Although initial results are promising, there are currently no randomized controlled trials comparing the results of EUS-CLE with the gold-standard test, i.e., surgical histology. Additionally, it is not possible to perform additional testing, such as immunohistochemical staining with EUS-CLE [108].

Over the years, researchers have tried to identify an ideal biomarker for pancreatic cancer, as cystic fluid analysis has a low diagnostic yield of around 50% [110]. A breakthrough in this search spanning over many decades is believed to be next-generation sequencing (NGS), wherein the cystic fluid from PCLs is analyzed for DNA mutations in an attempt to differentiate the type of pancreatic lesion [111]. For example, literature reports that the sensitivity and specificity of NGS for mutations of guanine nucleotide-binding protein-alpha subunit (GNAS), mitogen-associated protein kinase (MAPK), and KRAS for diagnosing mucinous PCLs is 90% and 100%, respectively, whereas multiple endocrine neoplasia 1 (MEN 1) and loss of heterozygosity (LOH) genes are associated with pancreatic neuroendocrine tumors with a combined sensitivity and specificity of 71% and 100%, respectively [111,112]. Additionally, cystic fluid NGS has also revealed that the loss of function mutation of the Von Hippel–Lindau gene was associated with serous cystadenoma with a sensitivity and specificity of 71% and 100%, respectively [112]. Furthermore, NGS for tumor protein 53, SMAD4, mammalian target rapamycin, and CTNNB1 genes are useful in identifying advanced neoplasia when combined with GNAS/MAPK mutation with a sensitivity and specificity of 88% and 98%, respectively [112]. Hence, NGS is emerging as a vital tool in establishing a diagnosis of pancreatic cancer as it allows clinicians to make an informed decision and plan the next steps in management.

## 10. Artificial-Intelligence-Augmented Endoscopic Ultrasound

Artificial intelligence (AI) involves the utilization of computer algorithms to analyze large datasets to identify patterns or make predictions [113,114]. In recent years, it has gained immense popularity in healthcare as it aids clinicians in image recognition and helps in complex clinical decision making [113,114]. Machine learning, a subtype of AI used in EUS, consists of two learning models, namely supervised and unsupervised learning. Supervised learning involves the use of labeled data to train the AI algorithm to recognize patterns in EUS images [115]. However, in unsupervised learning, the input data are unlabeled, and the AI model works to discover specific patterns and relationships within the data [115]. Furthermore, utilizing automatic segmentation with these AI models enables better visualization of the target area by identifying and separating different structures in EUS images [116,117].

AI-guided EUS-FNA has shown promising results in numerous studies. AI-enabled automatic visual inspection has proven to be helpful in rapid onsite tissue evaluation by indicating specific areas that are highly likely to indicate tumor cells in patients with pancreatic ductal adenocarcinoma with a sensitivity, specificity, and accuracy of about 80% [118,119]. Jiang et al. showed that the accuracy of AI was 99.6% in differentiating low- versus high-grade neoplasia, and Nuon et al. and Machicado demonstrated accuracies of 83% and 82% for AI models in differentiating mucinous cystic neoplasm versus serous cystadenocarcinoma and low versus high-grade dysplasia in intrapapillary mucinous neoplasm, respectively [120,121,122]. However, these findings should be interpreted in light of the fact that these studies were limited by a small sample size, usually from a single center. Generalizability and reproducibility of the results need to be demonstrated in large prospective multi-center studies or randomized controlled trials.

The integration of AI models in EUS has vast potential for improving the training of personnel, diagnostic accuracy, tumor grading, tumor staging, and determination of prognosis in patients with pancreatic cancer [123]. This can lead to significantly improved patient outcomes and a potential reduction in the number of repeat procedures as a result of non-diagnostic biopsies [123].

## 11. Conclusions

EUS has proven to be an essential tool for not only establishing an accurate diagnosis of pancreatic cancer but also differentiating it from other pancreatic lesions due to its ability to provide in-depth characterizations of these lesions. Compared to conventional cross-sectional imaging such as CT scan, MRI, or abdominal ultrasound, EUS has proven higher sensitivity, specificity, and diagnostic accuracy for diagnosing pancreatic cancer and characterizing other pancreatic lesions. Over the last few decades, there have been significant advancements in EUS, which have enabled therapeutic endoscopists to provide pancreatic cancer patients with a higher quality of care. New EUS modalities such as CE-EUS and EUS elastography complement each other by improving the characterization of pancreatic lesions. Additionally, with EUS-FNA, therapeutic endoscopists can obtain tissue samples from the lesion or surrounding lymph nodes in a minimally invasive fashion for a highly accurate tissue diagnosis, cancer staging, and immunohistological evaluation. Innovation in EUS with EUS-CLE, NGS of cystic fluid, and AI-augmented EUS models are expected to further revolutionize the field. However, additional large multi-center studies and randomized controlled trials are still needed to establish the validity of these new endoscopic techniques in the diagnosis and management of pancreatic cancer.

## Figures and Tables

**Table 1:** Classifications of pancreatic lesions.

Non-neoplastic cysts	Pseudocyst Simple/congenital cyst Retention cyst Mucinous non-neoplastic cyst Enterogenous cyst Periampullary duodenal wall cyst Endometrial cyst
Neoplastic cysts	**Mucinous cystic lesions** Intraductal papillary mucinous neoplasm Mucinous cystic neoplasm Serous cystic neoplasm **Non-mucinous cystic lesions** Serous cystadenocarcinoma Solid pseudopapillary neoplasm Cystic neuroendocrine neoplasm Acinar cell cystic neoplasm Cystic hamartoma Cystic teratoma Cystic pancreatoblastoma Ductal adenocarcinoma with cystic degeneration
Other neoplastic lesions	Pancreatic adenocarcinoma Acinar cell carcinoma Neuroendocrine tumors Lymphomas Metastases

**Table 2:** Characteristic features of various commonly encountered pathologies in endoscopic ultrasound.

Type of Lesion	Features in Endoscopic Ultrasound	Fluid Carcinogenic Embryogen	Fluid Amylase
Pancreatic Pseudocyst [37,38,39]	Anechoic, well-circumscribed, round or oval lesion, absence of septations and mural nodules	Low	High
Intraductal Papillary Mucinous Neoplasm [38,40]	Macrocystic-type lesion with occasional parenchymal changes and communication with pancreatic duct	High	High
Serous Cystadenoma [38,40]	Multiple microcysts (<3 mm) in a cystic lesion, possible honeycomb-like appearance, no ductal communication	Low	Low
Mucinous Cystic Neoplasm [38,41]	Cysts with septations of variable thickness, visible wall with occasional peripheral calcifications, no ductal communication	High	Low
Solid Pseudopapillary Neoplasm [38]	Mixed solid-cystic well-demarcated tumor	Low	Low
Pancreatic Adenocarcinoma [26,42,43]	Heterogenous, hypoechoic mass with an irregular border	High	Variable

**Table 3:** Indications for endoscopic ultrasound in the evaluation of pancreatic cystic lesions.

Guidelines	Indications for Endoscopic Ultrasound
Kyoto Guidelines for IPMN (2024)	The presence of any of the following “worrisome features”: Clinical:
	A. Acute pancreatitis B. Elevated serum CA 19-9 C. New onset or acute exacerbation of diabetes within the past year Imaging: A. Cyst size ≥3 cm B. Enhancing mural nodule <5 mm C. Thickened/enhancing cyst walls D. Main pancreatic duct ≥5 mm and <10 mm E. Abrupt change in caliber of the pancreatic duct with atrophy F. Lymphadenopathy G. Cystic growth rate ≥2.5 mm/year
European Evidence-Based Guidelines (2018)	Lesions with concerning features
	A. Growth rate >5 mm/year B. Elevated serum CA 19-9 (≥37 U/mL) C. Main pancreatic duct dilatation (5–9.9 mm) D. Cyst diameter ≥40 mm E. New onset of diabetes mellitus F. Acute pancreatitis G. Enhancing mural nodule <5 mm
American College of Gastroenterology (2018)	If any of the following present:
	-PD ≥ 5 mm -IPMN or MCN ≥ 3 cm -Change in PD caliber with upstream atrophy -Size increase of ≥ 3 mm/year during surveillance -Jaundice due to cyst -Pancreatitis due to cyst -Presence of a mural nodule or solid component
International Consensus (2017)	If any of the following present:
	-Pancreatitis due to cyst -Cyst size ≥ 3 cm -Enhancing mural nodule < 5 mm -Thickened/enhancing cyst walls -PD 5–9 mm -Abrupt change in diameter of PD with distal pancreatic atrophy -Lymphadenopathy -Elevated CA 19-9 -Rapid growth of cyst (>5 mm/2 years)
American Gastroenterological Association (2015)	≥2 high-risk features -Cyst size ≥ 3 cm -Pancreatic Duct Dilatation -Presence of a solid component
